# Immunosuppression after pediatric liver transplantation may lead to early and prolonged acute thymic involution: findings from a pilot longitudinal study

**DOI:** 10.3389/fimmu.2026.1864634

**Published:** 2026-06-26

**Authors:** Guillermo Costaguta, Brenda Dinatale, Itauá Leston Araujo, Wilson Savino, Fernando Alvarez, Oscar Bottasso, Ana Rosa Perez, Florencia Belén González, Alejandro Costaguta

**Affiliations:** 1 Pediatric Gastroenterology, Hepatology, and Nutrition, CHU de Quebec-Université Laval, Quebec, QC, Canada; 2 Research Centre of CHU de Quebec-Université Laval, Quebec, QC, Canada; 3 Instituto de Inmunología Clínica y Experimental de Rosario (IDICER-CONICET-UNR), Rosario, Argentina; 4 Facultad de Ciencias Médicas, Universidad Nacional de Rosario, Rosario, Argentina; 5 Laboratory on Thymus Research and National Institute of Science and Technology on Neuroimmunomodulation, Oswaldo Cruz Institute, Oswaldo Cruz Foundation, Rio de Janeiro, Brazil; 6 Pediatric Gastroenterology, Hepatology and Nutrition of CHU Sainte-Justine, Montreal, QC, Canada; 7 Department of Pediatrics, University of Montreal, Montreal, QC, Canada; 8 Liver Transplantation Unit, Sanatorio de Niños de Rosario, Rosario, Argentina

**Keywords:** immunosuppression, liver transplantation, recent thymic emigrants, sjTRECs, T-cell receptor excision circles, thymic output, thymus

## Abstract

Thymopoiesis plays a critical role in shaping the peripheral T-cell repertoire during childhood. Following pediatric liver transplantation, patients receive intensive immunosuppressive therapy, which may disrupt thymic function; however, thymic involvement after solid organ transplantation remains poorly understood. This prospective study aimed to assess the impact of immunosuppressive therapy on thymic function and morphology in pediatric first-time liver transplant recipients over a one-year follow-up period. The primary endpoint was thymic output, quantified by sjTRECs. Secondary endpoints included intrathymic proliferation (sj/βTREC ratio), recent thymic emigrants (RTEs; CD3^+^CD4^+^CD45RA^+^CD31^+^), and thymic morphology. All parameters were assessed at baseline (pre-transplant) and at 1, 3, 6, and 12 months post-transplantation. Twelve patients were included. The primary endpoint -thymic output measured by sjTRECs- declined significantly over the 12-month follow-up (β=-732.1, p<0.01). Among secondary endpoints, the sj/βTREC ratio, showed a significant reduction after 3 months post-transplant. The frequency of recent thymic emigrants (RTEs) showed a decreasing trend that did not reach statistical significance, although RTEs correlated positively with sjTREC levels. Thymic size assessed by ultrasound decreased significantly at 3 months, with partial recovery thereafter. Taken together, our findings provide preliminary evidence of early and persistent changes in markers of thymic function following immunosuppression after pediatric liver transplantation. Although the small sample size and exploratory design of this pilot study warrant cautious interpretation, the statistically significant changes observed in the primary endpoint justify the need for further controlled studies to better characterize the impact of immunosuppressive therapy on thymic recovery and long-term immune reconstitution in pediatric transplant recipients.

## Introduction

In early life, the immune system is undergoing active development and is characterized by high thymic activity, which plays a central role in shaping the T-cell repertoire. In this context, pediatric patients—especially those transplanted at a very young age—may be particularly vulnerable to immune perturbations, potentially linked to this heightened thymic function, mainly when considering their immunosuppressive interventions.

Recent advancements in liver transplantation have greatly enhanced both graft and patient survival rates, particularly in pediatric patients, making it a viable treatment option for many previously life-threatening liver diseases (1). In the pediatric population, liver transplantation is a well-established procedure. Data from Argentina showed that nearly 25% of the approximately 500 liver transplants performed in 2023 involved patients under 18 years of age (2). Despite these progresses, immunosuppression continues to pose significant challenges, particularly for children. Transplanted patients, especially those who undergo transplantation early in life, are at an increased risk of opportunistic infections, alloimmune hepatitis, chronic rejection, and both immediate and long-term adverse effects of immunosuppressive therapy. Notably, up to two-thirds of late deaths following pediatric liver transplantation are associated with complications arising from immunosuppression (3, 4). In this context, it is crucial to develop immunosuppression protocols that both prevent rejection and minimize side effects. Pediatric patients, especially those who undergo transplantation before the age of two, may have a greater ability to develop graft tolerance (5, 6). This potential is probably linked to the increased thymic activity characteristic of that age, a period marked by vigorous T-cell development (7).

Natural thymic involution, characterized by the replacement of stromal tissue with adipose tissue, typically begins during adolescence and continues to extend and deepen throughout adulthood, progressively impacting immune function. This process results in decreased production of naïve T-cells and a predominance of memory T-cells in circulation (8, 9). The thymus, however, is highly sensitive to physiological stress and external factors, including infections, malnutrition, and pharmacological interventions, such as immunosuppression, which can cause acute thymic involution (ATI), a rapid reduction in thymic cellularity (7, 10, 11).

In this context, the use of immunosuppressive therapies after transplantation like calcineurin inhibitors (such as cyclosporine or tacrolimus) or basiliximab during a critical window of immune development, such as childhood, may interfere with normal thymic function and consequently impact upon thymic T-cell homeostasis and long-term immune regulation. In addition, clinical and experimental studies in both humans and animals indicate that exposure to high levels of glucocorticoids, whether pharmacological/immunosuppressive or stress-induced, leads to reduced thymic output and naïve T-cell depletion, consistent with glucocorticoid-induced thymic atrophy (12–17). However, thymic involvement following solid transplantation remains poorly understood/explored. This could be partly because thymic activity is difficult to assess, particularly in children. In recent years, the quantification of T-cell receptor excision circles (TRECs) has been used as a reliable tool to evaluate thymic activity from small samples of peripheral blood. Thymic T-cell maturation involves the rearrangement of T-cell receptor (TCR) α and β chains, leading to the formation of episomal DNA fragments (TRECs), specifically signal joint TRECs (sjTRECs) and beta TRECs (βTRECs). These fragments are stable and do not replicate, thus becoming diluted with each cell division. They are not easily degraded, and their presence unequivocally indicates that the cells originate from the thymus (18). SjTRECs are widely used to assess thymic activity and export, as a higher number of cells emigrating from the thymus results in a greater number of sjTRECs detected in circulation, making them widely used as surrogate markers of thymic output of naïve RTE (19–21). Additionally, a more accurate assessment of thymic function can be achieved by the sj/βTRECs ratio, which reflects intrathymic proliferation by indicating the number of divisions occurring among CD4^-^CD8^-^double negative (DN) and CD4^+^CD8^+^ double positive (DP) developing thymocytes (21, 22).

In this study, we aimed to determine whether induction immunosuppression in pediatric liver transplantation leads to accelerated thymic involution, as indicated by a reduction in thymic output, proliferative capacity, and thymic size, over a follow-up period of up to one year post-transplantation Understanding these effects may guide the development of immunosuppressive strategies that maintain thymic activity, potentially improving graft acceptance and long-term patient outcomes.

## Methods

### Study population

This study involved 12 pediatric patients, who underwent their first liver-only transplantation at the Liver Transplant Unit of Sanatorio de Niños (Rosario, Argentina) between January 2021 and December 2024. Inclusion criteria were (1): age between 0 and 18 years (2); first liver-only transplantation performed at our unit during the study period; and (3) liver transplantation for any underlying liver disease, without etiological restrictions. Exclusion criteria were (1): previous or concurrent multi-organ transplantation (2); congenital or acquired immunodeficiency, including conditions known to affect thymic development or function (e.g., DiGeorge syndrome); (3) use of immunosuppressive therapy for conditions other than liver transplantation within 3 months prior to enrollment; and (4) death before enrollment or completion of follow-up. Patients were monitored prospectively for 12 months following transplantation. Visits were scheduled weekly during the first month, biweekly in the second and third months, and monthly thereafter. At each visit, clinical assessments, laboratory tests, and imaging studies were performed. During the follow-up period, we systematically documented patient and graft survival, as well as bacterial, CMV, EBV, and other viral infections, alongside episodes of acute cellular rejection. Most transplants were due to acute liver failure and biliary atresia. All participants completed the scheduled follow-up assessments. Missing data were limited and generally consisted of a single missed evaluation at one time point for a small number of patients, reflecting the practical challenges of conducting repeated assessments in a pediatric transplant population. This was mainly related to the challenges of conducting repeated evaluations in a pediatric transplant population, including missed visits, clinical instability, hospitalization, logistical constraints, or unavailable biological samples and imaging studies.

The characteristics of the study population at 0-, 3-, 6-, and 12-month post-transplantation are detailed in **Table 1**.

**Table 1:** Primary diagnosis, and 12-month outcomes and complications after liver transplantation.

Primary diagnosis	Number of cases				
Acute liver failure (indeterminate)	4				
Biliary atresia	3				
Autoimmune hepatitis	2				
Caroli’s syndrome	1				
Wilson’s disease	1				
Gestational alloimmune liver disease	1				
TOTAL:	12				
Variables	Time-points after LT (months)				
	1	3	6	12
Patient survival	12/12	12/12	12/12	12/12
Graft survival	10/12	10/12	10/12	10/12
Bacterial infections	1/12	5/12	6/12	6/12
CMV infections	6/12	7/12	7/12	8/12
EBV infections	0/12	2/12	3/12	4/12
Other viral infections	1/12	1/12	1/12	1/12
Acute cellular rejection	2/12	4/12	6/12	6/12
Autoimmune phenomena	1/12	1/12	2/12	2/12

All patients received a standardized induction immunosuppression regimen that included methylprednisolone, tacrolimus, and basiliximab. After induction, all of them received corticosteroids at a dosage of 2 mg/kg until transaminase levels normalized, with a gradual tapering to a physiological dose (**Supplementary Material 1**).

### Study endpoints

The primary endpoint was the longitudinal change in thymic output, assessed by quantification of signal-joint T-cell receptor excision circles (sjTRECs) in peripheral blood. sjTRECs are widely recognized as one of the most reliable and extensively validated surrogate markers of thymic function and recent thymic emigrant production. Thymic output was expressed as the absolute number of sjTREC copies per 150,000 cells and evaluated from baseline (pre-transplantation) to 12 months after liver transplantation.

Secondary endpoints included: 1) the frequency of recent thymic emigrants (RTEs), defined as CD3^+^CD4^+^CD45RA^+^CD31^+^ T cells and measured by flow cytometry; 2) the sj/βTREC ratio, used as a surrogate marker of intrathymic proliferation and 3) the thymic area (mm²), assessed by ultrasonography as a morphological correlate of thymic status.

All endpoints were evaluated at baseline (pre-LT) and at 1, 3, 6, and 12 months after transplantation.

### Thymic function analysis

To evaluate thymic functional capacity in terms of thymic output and intrathymic proliferation, sjTRECs and βTRECs were quantified at 0, 1, 3, 6, and 12 months after immunosuppressive treatment initiation. Values of β.1 TRECs correspond to the sum of β.1.1 through β.1.6, while β.2 TRECs represent the sum of β.2.1 through β.2.4. Total βTRECs are calculated as the combined sum of β.1 and β.2 TRECs. The quantification of TRECs was performed using dried blood spot samples, 50 μl of fresh whole blood was deposited as drops onto a certified filter paper and left to dry for 1 h at room temperature. The samples were then stored at -80 °C until processing. DNA was extracted using a commercial DNA extraction kit, following the detailed protocol provided for this type of sample (QIAamp DNA Mini Kit, Qiagen, Germany). The yield from DNA from six 3 mm diameter dried blood spots was approximately 5 ng/µL. To increase the sensitivity of the method, 50 ng of DNA was pre-amplified using conventional PCR with primers specific for sj and βTRECs. In parallel, amplification of the albumin gene was used as quality and quantity control for genomic DNA in each sample. The principle of the assay for quantifying TRECs was based on a qPCR technique using TaqMan probe technology (23). Briefly, 5 µL of DNA were amplified in a final reaction volume of 20 µL containing 10 µL of TaqMan^®^ Universal PCR Master Mix (Applied Biosystems™), 1 µL of a 20× primer/probe mix specific for each TRECs, 1 µL of a 20× primer/probe mix for the albumin gene, and 3 µL of nuclease-free water (See Supplementary Material 2). Cycling conditions included an initial denaturation at 95 °C for 5 minutes, followed by 40 cycles of 95 °C for 15 seconds and 60 °C for 1 minute. Amplification and detection were performed using the QuantStudio 3 Real-Time PCR System (Applied Biosystems™, Thermo Fisher Scientific), according to the manufacturer’s specifications. Fluorescence data analysis and cycle threshold (Ct) determination were carried out using the instrument’s software. Absolute quantification of TRECs and the albumin gene was performed by extrapolation from a standard curve generated using serial dilutions of a plasmid containing one copy of each TREC and albumin genes. This plasmid was provided by the Laboratório de Pesquisas sobre o Timo (LPT-FIOCRUZ, Brazil). TRECs were reported as TRECs per 150,000 cells.

Additionally, as a complementary measure to sjTREC and sj/βTREC quantification, the presence of RTE in circulation was analyzed by flow cytometry. Circulating cells with the CD3^+^CD4^+^CD45RA^+^CD31^+^ phenotype contained the highest levels of sjTRECs, indicating that they have recently emigrated from the thymus. Therefore, we evaluated the percentage of this population in the blood of patients 0, 1, 3, 6, and 12 months after the initiation of immunosuppressive treatment. To do this, a 100 µl aliquot of fresh blood was incubated with the following antibodies for 30 minutes: anti-CD3/APC-Cy7 (Invitrogen #MA5-38705), anti-CD4/APC (BD Pharmingen #555349), anti-CD45RA/FITC (BD Pharmingen #555488), and anti-CD31/PE-Cy7 (Invitrogen #25-0319-42). Next, red blood cells were lysed by adding a lysis solution (0.15 M NH_4_Cl, 10 mM KHCO_3_, 0.1 mM Na_2_EDTA, pH 7.3). After washing, the white blood cells were resuspended in 4% formaldehyde fixation solution for 1h. The sample was then washed and resuspended in 1X phosphate-buffered saline (PBS). Samples were analyzed using a BD FACSAria II flow cytometer (Becton Dickinson, USA). During the analysis, dead cells were excluded based on light scatter properties (forward scatter [FCS] and side scatter [SSC]), and lymphocyte populations were selected based on size and complexity. A minimum of 1.10^5^ events was acquired for each sample. Frequency analyses were performed using the FlowJo v10 software (Becton Dickinson, USA).

### Thymic morphology analysis

Thymic size was assessed via ultrasonography at 0-, 1- 3-, 6-, and 12-months post-transplantation, by the same experienced operator following standardized protocols (24). Baseline measurements, taken immediately before transplantation, served as a reference to calculate changes in thymic size over time. Due to the thymus’s varying shapes, both anteroposterior and longitudinal dimensions were measured and multiplied to estimate the approximate surface area in square millimeters. The median surface area at each time point was calculated, and changes over time were analyzed using Brown-Forsythe and mixed-effects analysis to pinpoint when the means became significantly different.

### Statistical analysis

Descriptive statistics were utilized to summarize clinical, biochemical, and imaging data. For paired group comparisons, Friedman’s non-parametric test was applied. In contrast, the Kruskal–Wallis tests were used for comparisons between independent groups, with *post-hoc* analysis conducted when necessary. The Wilcoxon signed-rank test was used to compare two paired datasets. Categorical variables were analyzed using either the chi-squared or Fisher’s exact test. Longitudinal changes in thymic parameters were analyzed using Generalized Estimating Equations (GEE) to account for repeated measurements and within-subject correlations over time. Missing values were not imputed, and longitudinal analyses were performed using GEE, which allows the inclusion of participants with partially missing repeated-measures data, making use of all available observations. Given the well-established influence of age on thymic function, age was included as a covariate in the model to adjust for its potential confounding effect. This approach allowed the evaluation of temporal changes in thymic morphology and function while accounting for both within-subject correlations and age-related variability. Correlation analyses were conducted using Spearman’s rank correlation. Statistical significance was determined at p < 0.05. Analyses were performed using GraphPad Instat 4.0 and 9.5 (GraphPad, California, USA).

## Results

### Longitudinal assessment of TRECs and RTEs following liver transplantation.

**Figure 1:**
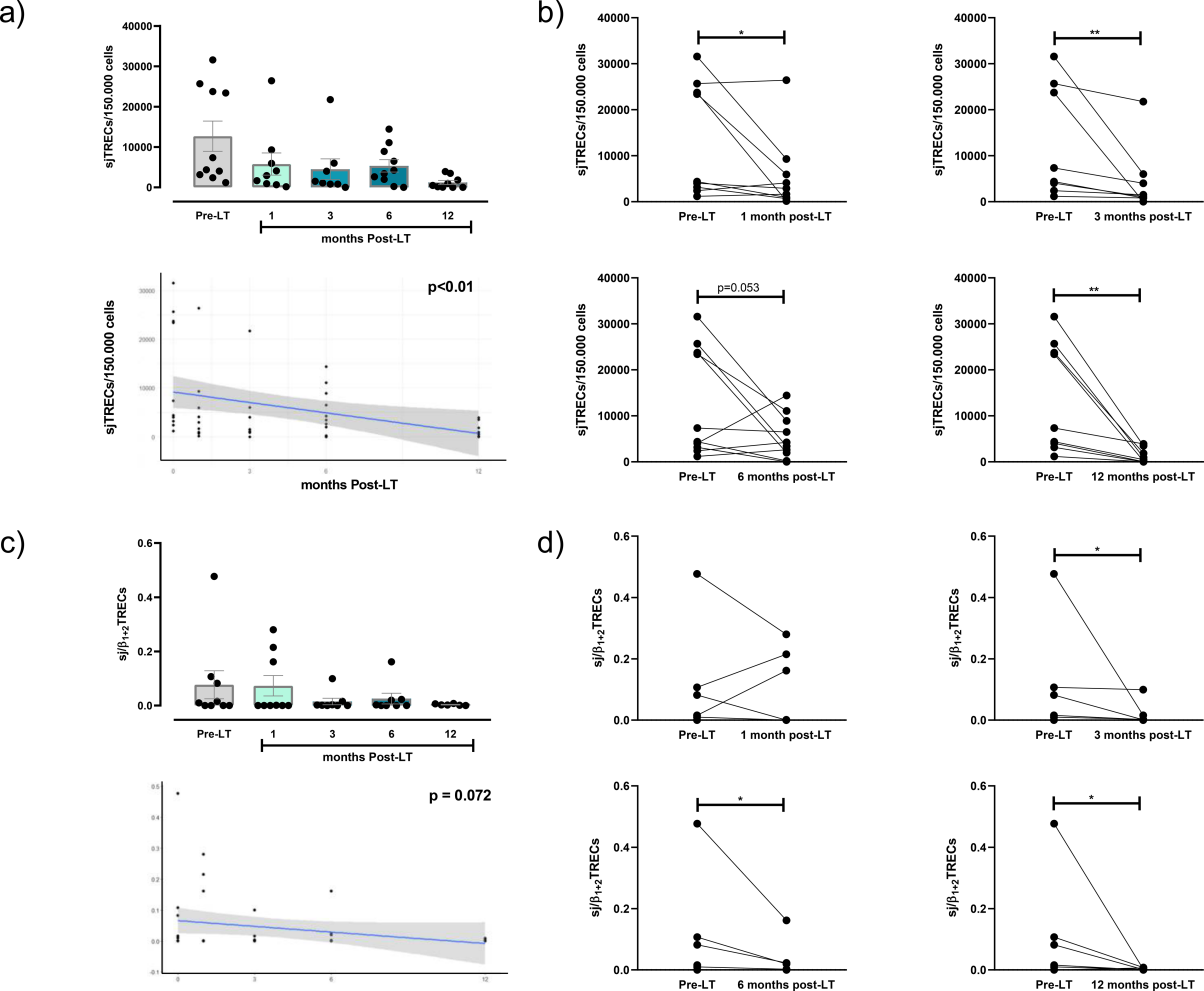
Longitudinal changes in thymic output following liver transplantation. **(A)** Quantification of sjTRECs (copies/150.000 cells) at different time points: pre-liver transplantation (LT) and 1, 3, 6, and 12 months post-LT. In the upper panel, data are shown as individual values as well as the mean with SEM. The lower panel shows the longitudinal trend with linear regression analysis. **(B)** Paired comparisons of sjTREC levels between Pre-LT and each post-transplant time point (1, 3, 6, and 12 months). Each line represents an individual patient. **(C)** sj/β_1+2_ TREC ratio at the same time points, shown as individual values as mean with SEM. The lower panel depicts the longitudinal trend with linear regression analysis. **(D)** Paired comparisons of sj/β_1+2_ TREC ratio between Pre-LT and each post-transplant time point. Each line represents an individual patient. P values are indicated in the figure (*p < 0.05, **p < 0.01).

**Figure 2:**
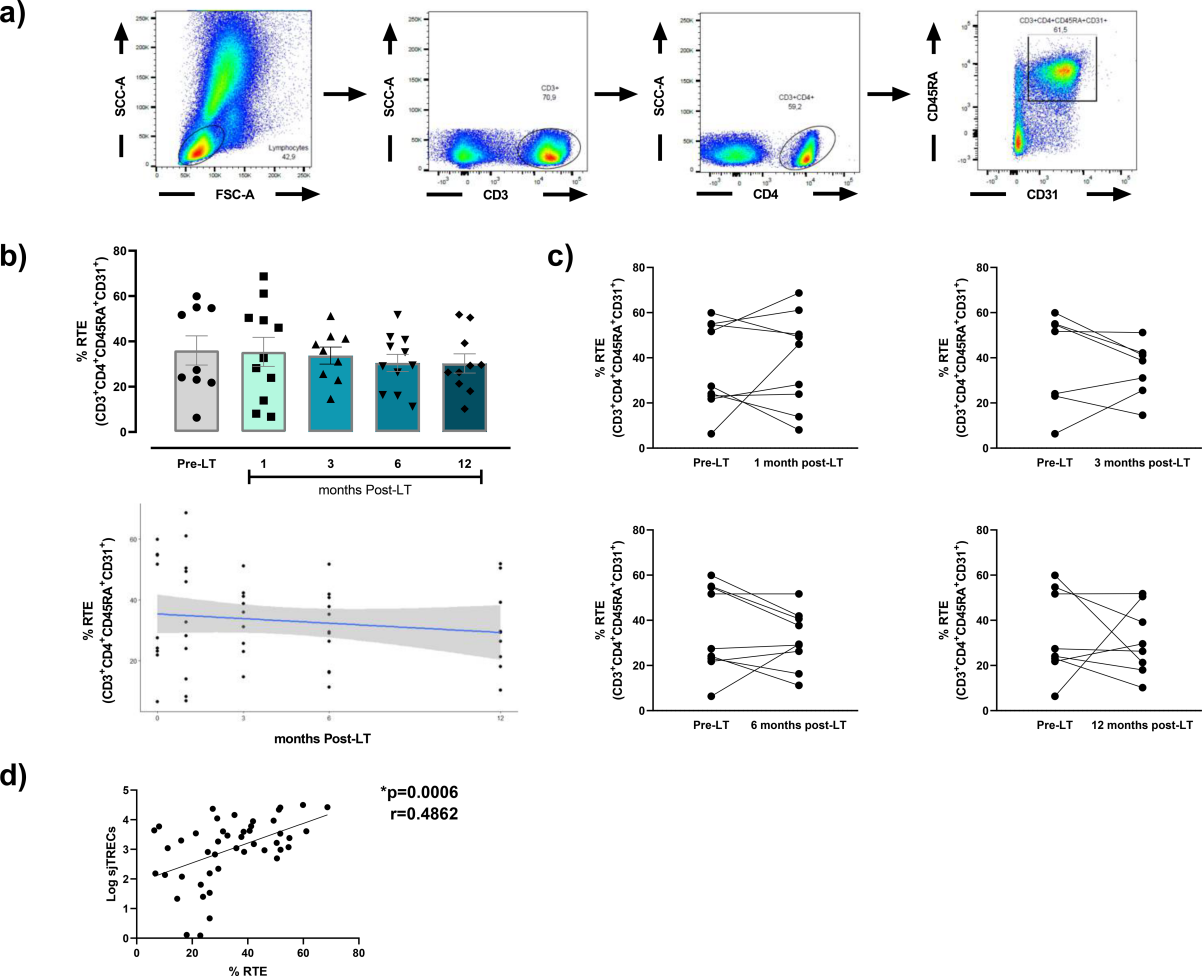
Longitudinal evaluation of recent thymic emigrants (RTEs) following liver transplantation. **(A)** Representative flow cytometry gating strategy used to identify RTEs, defined as CD3^+^CD4^+^CD45RA^+^CD31^+^ T cells. Lymphocytes were first gated based on FSC-A and SSC-A, followed by sequential gating on CD3^+^ and CD4^+^ populations, and finally identification of CD45RA^+^CD31^+^ cell subsets. **(B)** Frequency of RTEs (% CD3^+^CD4^+^CD45RA^+^CD31^+^) at pre-liver transplantation (Pre-LT) and at 1, 3, 6, and 12 months post-LT. Data are shown as individual values as mean with SEM. The lower panel shows the longitudinal trend with linear regression analysis. **(C)** Paired comparisons of RTE frequencies between Pre-LT and each post-transplant time point (1, 3, 6, and 12 months). Each line represents an individual patient. **(D)** Correlation between RTE frequency and sjTREC levels (log-transformed). The p-value is indicated in the figure.

Changes in thymic output were evaluated through serial measurements of sjTRECs as primary endpoint (**Figures 1a, b**) and recent thymic emigrants (RTEs; **Figure 2**) as secondary endpoint at multiple time points following liver transplantation. The secondary endpoint sj/β_1+2_ TRECs ratio was also used as a surrogate marker of intrathymic proliferation (**Figures 1c, d**). A significant decline in sjTREC levels was observed over time (β = -732.1, p < 0.01; **Figure 1a**), as demonstrated by Generalized Estimating Equations analysis accounting for within-subject correlation. Paired analyses comparing Pre-liver transplantation (LT) values with each post-LT time point reveal a consistent reduction in sjTREC levels after transplantation (**Figure 1b**). This decrease reaches statistical significance at 1, 3, and 12 months post-LT (**p < 0.01), while a trend is observed at 6 months (p = 0.053). Individual trajectories highlight that most patients experience a marked decline in thymic output after liver transplantation.

Although a clear downward trend is observed over time for the sj/β TREC ratio, this change does not reach statistical significance (p = 0.072) when the overall population is analyzed, suggesting a modest impact on thymic proliferative activity (**Figure 1c**). Nevertheless, paired comparisons of sj/β TREC ratios between pre-LT and post-LT time points show a significant reduction at later time points, particularly at 3, 6, and 12 months post-LT, suggesting impaired intrathymic proliferation following transplantation (**Figure 1d**). Overall, these data demonstrate a sustained decrease in thymic output, as measured by TRECs, after LT, with evidence of both reduced recent thymic emigrants and altered thymic proliferative dynamics over time.

**Figure 3:**
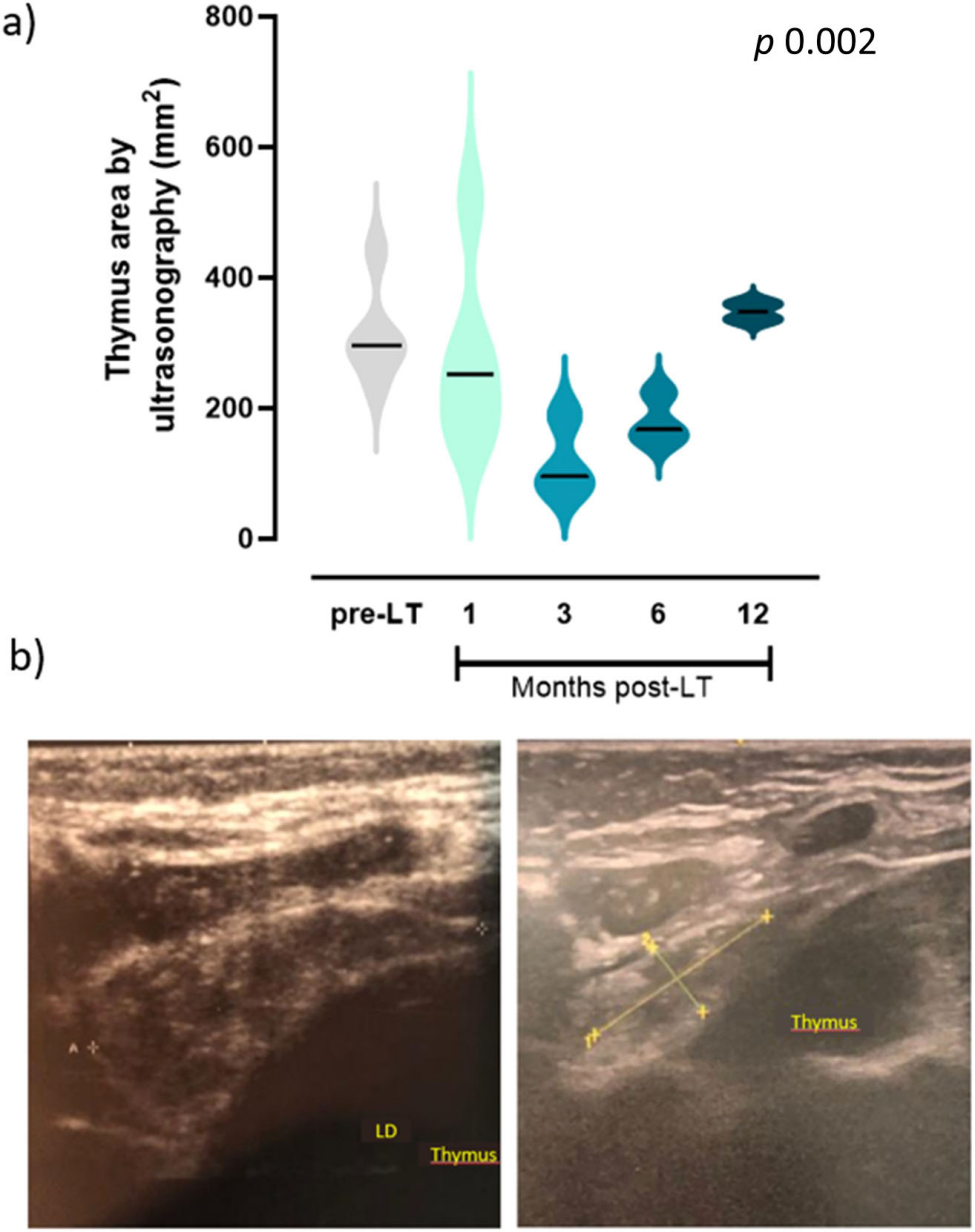
Ultrasound assessment of thymic size dynamics following liver transplantation. **(A)** Thymus area (mm²) measured by ultrasound at pre-liver transplantation (Pre-LT) and at 1, 3, 6, and 12 months post-LT. Data are presented as violin plots with median values. A statistically significant difference across time points was observed (p = 0.002). **(B)** Representative ultrasound images of the thymus. The thymic area was identified and measured using anatomical landmarks; the thymus is indicated, and measurement axes are shown.

Thymic output was also assessed by quantifying through flow cytometry RTEs within the CD3^+^CD4^+^CD45RA^+^CD31^+^ T cell compartment. A representative gating strategy is shown in **Figure 2a**. At the group level, the frequency of RTEs did not show significant differences over time when comparing pre-transplant values with those at 1, 3, 6, and 12 months post-transplantation (**Figure 2b**; β = -0.314, p = 0.48). Longitudinal paired analyses revealed heterogeneous dynamics, with patients showing either increases or decreases in RTE frequencies at different time points (**Figure 3c**).

However, a significant positive correlation was found between RTE frequency and thymic output measured by sjTRECs (log-transformed), supporting the validity of RTEs as a surrogate marker of thymic function (**Figure 2d**), although TRECs appear to be more sensitive and show less variability as markers of thymic export.

### Changes in thymic size after liver transplantation

Among the 12 patients undergoing transplantation, only five had their thymus size fully assessed at all time points by ultrasound, with complete data available from pre-LT to 12 months post-LT (**Figure 3a**). In four patients, the gland was not visible at pre-LT but was detected at some or most subsequent time points, precluding a comprehensive analysis of changes in gland area. In the remaining three patients, the gland was never visualized. A statistically significant difference was observed across time points (pre-LT median 296 mm²; post-LT: 1 month 252 mm², 3 months 96 mm², 6 months 178.5 mm², 12 months 348 mm²; p = 0.002). *Post hoc* multiple comparisons revealed that the difference became significant starting at 3 months post-LT.

## Discussion

Thymic function peaks in infancy and progressively declines after puberty due to natural thymic involution, characterized by atrophy, loss of thymocytes, and replacement of thymic epithelial cells by adipose tissue. This leads to reduced naïve T-cell output and TCR diversity, with a shift toward memory T cells, becoming evident around 30–40 years of age (25, 26). However, under certain pathological conditions, the thymus can experience an ATI, characterized by increased death of thymocytes and thymic epithelial cells. Unlike normal thymic senescence, this acute involution may be reversible if the causative agent is removed (15, 27, 28). Most of these conditions are of infectious origin, including bacterial (29), viral (30), parasitic (31), or fungal infections (10, 32). Nonetheless, various other stimuli can accelerate double-positive apoptosis, such as immunosuppressive agents. Among these, glucocorticoids are one of the most extensively studied (14–17). Therefore, the distinction between normal physiological involution and ATI becomes particularly relevant in the context of solid organ transplantation, where immunosuppressive protocols commonly involve the administration of high-dose glucocorticoids (33).

However, most of the data on the thymus and immune reconstitution following immunosuppression derive from studies of allogeneic hematopoietic stem cell transplantation (HSCT). These studies have shown that reduced pre-transplant sjTREC levels, as a measure of thymic output, are associated with an increased risk of acute graft-versus-host disease (aGvHD), particularly grades II–IV (18, 19, 34, 35). Similarly, low sjTREC levels correlate with a higher incidence of bacterial infections and CMV reactivation (34). Consistent with these findings, diminished sjTREC levels have also been linked to increased infectious complications in myeloma patients undergoing autologous HSCT (36). Together, these results underscore the critical role of pre-transplant immune competence. Importantly, multivariate analyses have identified recipient thymic function as a key pre-transplant determinant of HSCT outcomes, emphasizing the thymus as a pivotal regulator of immune reconstitution and clinical prognosis (34). In another study, 210 patients were analyzed for sjTREC levels during the first 24 months after transplantation. Patients with TREC levels above the median at 3 months exhibited superior overall survival (80% vs. 56%) and lower transplantation-related mortality (7% vs. 21%). These findings strongly support the use of TREC measurement as part of the standard repertoire of immunological monitoring after HSCT (37).

In contrast, there is limited information regarding thymic function and transplant outcomes in the context of solid organ transplantation. Some studies have reported an association between low pre-transplant thymic function and increased mortality after renal transplantation; however, they do not address thymic involvement following transplantation under immunosuppressive therapy (38). In another study, thymic function was evaluated both before transplantation and during the first-year post-transplant, revealing a marked reduction in circulating naïve T cells driven by a decrease in recent thymic emigrants (RTEs), which was strongly associated with all-cause mortality (39).

This knowledge gap is particularly relevant in pediatric populations, where disruptions in thymic activity may have lasting consequences for immune competence and clinical outcomes throughout life. Most pediatric liver transplants are performed before the age of six, a period of peak thymic activity, raising important concerns about the impact of early, intensive immunosuppression on thymic function and immune development (40). However, thymic involvement following liver transplantation remains poorly understood, mirroring the broader scarcity of data on thymic function after solid organ transplantation in general. Therefore, in this pilot study, we aimed to non-invasively assess both the morphological and functional status of the thymus in a limited cohort of pediatric patients undergoing their first liver-only transplant. This evaluation was conducted prospectively for one year after transplantation using ultrasound imaging of the thymus, determining thymic output (sjTRECs and RTEs), and estimating the thymus’s capacity to expand its DN/DP populations (sj/βTREC ratio). Age is one of the main determinants of thymic function during childhood and was taken into consideration in the statistical analyses. However, given the limited sample size, the potential effects of other confounders, such as underlying disease characteristics, clinical status, nutritional factors, or individual differences in immunosuppressive exposure, could not be fully assessed. Furthermore, although the inclusion of an age-matched control group would have strengthened the study, obtaining repeated longitudinal thymic assessments in healthy pediatric subjects poses important ethical and logistical challenges. To partially address this limitation, each patient served as their own control, with pre-transplant measurements used as the individual baseline for comparison throughout follow-up.

In line with the development of an ATI, we observed that pediatric liver transplant recipients undergoing immunosuppression exhibited a significant reduction in sjTRECs and sj/βTREC ratio in peripheral blood, consistent with a potential decline in thymic output and intrathymic proliferation. This is somewhat supported by the findings of thymic size reduction. The gland’s surface significantly decreases immediately after transplantation, particularly between 1 and 3 months post-trasplant. However, in the later stages (6 and 12 months post-trasplant), there appears to be a trend toward recovery in size. These observations suggest that after the initial impact, characterized by high doses of immunosuppression, principally glucocorticoids, the thymus appears to gradually recover, although it does not fully return to normal even a year after transplantation. This apparent recovery is not reflected in sjTRECs or sj/βTREC ratio, which remains significantly decreased one-year post-transplantation. Importantly, previous studies have demonstrated that thymic size does not necessarily correlate with thymic function (41). Imaging-based assessments of thymic volume provide only a structural estimate and do not reliably reflect thymic output, as measured by sjTRECs quantification. Indeed, both clinical and experimental studies have reported discordance between thymic size and function, including conditions in which thymic enlargement occurs despite reduced TREC levels (42, 43). Together, these findings support the concept that morphological recovery of the thymus may not correspond to effective thymopoiesis, consistent with our observations.

In parallel, we also assessed RTEs by flow cytometry using CD31 as a marker. Although sjTREC levels and RTE frequencies were positively correlated, the proportion of RTEs remained relatively constant across the different time points analyzed. While CD31 is commonly used as a marker of RTEs, its expression may be partially retained under certain conditions following peripheral homeostatic proliferation (44). In the context of reduced thymic output, the naïve T-cell pool may be maintained through compensatory proliferation; if CD31 expression is transiently retained after such proliferation, this could result in TREC dilution without a proportional decrease in CD31^+^ T cells. Further studies are needed to address this question.

Beyond the potential effects of transplant-related immunosuppression on the thymus, growing evidence suggests that thymic health may have broader implications for long-term immune homeostasis and clinical outcomes. A recent large population-based study demonstrated that preserved thymic health in adults was associated with lower all-cause mortality and reduced risks of cardiovascular disease and cancer, supporting the concept that thymic function remains relevant throughout life (45). Similarly, radiological studies in patients with autoimmune diseases have showed a consistent pattern of accelerated thymic involution compared with healthy controls, suggesting that alterations in thymic structure and function may contribute to impaired immune tolerance and disease susceptibility (46). Furthermore, long-term immunological alterations have been described in children undergoing corrective cardiac surgery involving thymectomy, highlighting the potential consequences of thymic disruption during early life (47).

In conclusion, this pilot study provides preliminary evidence suggesting that thymic morphology and function may be altered during the first year of immunosuppression following pediatric liver transplantation. Although the reported changes in thymic markers are consistent with acute thymic involution, the small sample size and exploratory design warrant cautious interpretation of these findings. Nevertheless, these observations highlight a potentially underrecognized aspect of post-transplant immune reconstitution during a period of peak thymic activity, generating hypotheses worth testing. Larger, controlled studies are needed to confirm these findings, clarify the mechanisms involved, and assess their potential implications for long-term immune competence, thymic recovery, and clinical outcomes in pediatric solid organ transplant recipients.

## Data Availability

The raw data supporting the conclusions of this article will be made available by the authors, without undue reservation.
