# Supplementary material for: Immunosuppression after pediatric liver transplantation may lead to early and prolonged acute thymic involution: findings from a pilot longitudinal study
Source: Front Immunol. 2026 Jun 26;17:1864634. doi: 10.3389/fimmu.2026.1864634 (PMC13349776; doi:10.3389/fimmu.2026.1864634)
Supplement: Supplementary file 1 [file Table1.docx]

**Supplementary material 1: Immunosuppression protocol**

- Methylprednisolone: 10 mg/kg bolus IV at reperfusion in the operating room, continued with 2 mg/kg/day until 1normalization of aminotransferases and GGT, then switch to PO prednisone and taper 10-15% every 3 to 7 days until a maintenance dose of 3.75 mg/m^2^ PO. Switch to hydrocortisone, and taper slowly until recovery of suprarenal function, then withdraw (approximately between the third and fourth post-operative month)
- Basiliximab: Two separate IV doses on days 0 and 4. In <35 Kg: 10 mg/dose, in >35 Kg: 20 mg/dose
- Tacrolimus: From day 5, postoperative targeting of blood levels of 10–12 ng/mL during the first month. Taper to 4-5 ng/mL at six months
