# Supplementary material for: Immunosuppression after pediatric liver transplantation may lead to early and prolonged acute thymic involution: findings from a pilot longitudinal study
Source: Front Immunol. 2026 Jun 26;17:1864634. doi: 10.3389/fimmu.2026.1864634 (PMC13349776; doi:10.3389/fimmu.2026.1864634)
Supplement: Supplementary file 2 [file Table2.docx]

**Supplementary material 2**

| **Primer** | **Sequence** |
| --- | --- |
| sjTREC Forward | CACATCCCTTTCAACCATGCT |
| sjTREC Reverse | GCCAGCTGCAGGGTTTAGG |
| βTREC 1.1 | TGTCCTCCATCCTAGCCAGG |
| βTREC 1.2 | TCCGTCACAGGGAAAAGTGG |
| βTREC 1.3 | TGTCCCTGTGAGGGAAGAGTT |
| βTREC 1.4 | TGGACTTGGGGAGGCAGGA |
| βTREC 1.5 | CTCATAAAATGTGGGTCAGTGGA |
| βTREC 1.6 | TGAATCCAGGCAGAGAAAGG |
| βTREC 2.1 | CCAGCTAACTCGAGACAGGAA |
| βTREC 2.2 | GAACCCTGTTCTTAGGGGAGT |
| βTREC 2.3 | TGAGAGGGGCTGTGCTGAGA |
| βTREC 2.4 | AAGCGGGGGCTCCCGCTGAA |
| Db1 | TGTGACCCAGGAGGAAAGAAG |
| Db2 | GGACCAGCCCCAGAGAA |
| Albumin Forward | GCTGTCATCTCTTGTGGGCTGT |
| Albumin Reverse | ACTCATGGGAGCTGCTGGTTC |
| **Probe** | **Sequence** |
| sjTREC | FAM-ACACCTCTGGTTTTTGTAAAGGTGCCCACT-TAMRA |
| β1TREC | FAM-CAAAAACCTCTCTGGCGGTCCCAAC-TAMRA |
| β2TREC | FAM-CCCACCCAGCTCAGGGAATGCA-TAMRA |
| Albumin | FAM-CCTGTCATGCCCACACAAATCTCTCC-TAMRA |
